# A comparison of interspecific and intraspecific phenotypic variation in spectral signatures of ferns with robust versus uncertain species boundaries

**DOI:** 10.1007/s10265-026-01697-1

**Published:** 2026-03-09

**Authors:** Niksoney Azevedo Mendonça, Marise Helen Vale de Oliveira, Thaís Elias Almeida

**Affiliations:** 1 Departamento de Botânica, Programa de Pós-graduação em Biologia Vegetal, Centro de Biociências, Universidade Federal de Pernambuco, Av. Professor Moraes Rego, 1235, Recife, PE 50670-420 Brazil; 2 Programa de Pós-graduação em Botânica, Instituto Nacional de Pesquisas da Amazônia, Av. André Araújo, 2936, Manaus, AM 69067-375 Brazil

**Keywords:** Ferns, FT-NIR spectroscopy, *Microgramma*, Predictive models, Species limits, Systematics

## Abstract

Species delimitation methods based on macromorphology are often limited by phenotypic plasticity in plants. Fourier Transform near-infrared spectroscopy (FT-NIR) provides a promising alternative as a non-destructive technique that measures molecular vibrations (overtone and combination bands of C–H, N–H, and O–H bonds) from plant tissue exposed to near-infrared light (780–2,500 nm). We applied FT-NIR to the taxonomically challenging Scaly clade of *Microgramma* ferns (94 samples, eight species), including dimorphic and monomorphic taxa, to evaluate its diagnostic potential. Using multivariate models and cross-validation, we achieved 81–100% average identification accuracy. Well-defined species (e.g., *M. percussa*) reached 100% accuracy, while morphologically overlapping taxa showed lower accuracy, likely due to hybridization, introgression, or cryptic variation. Dimorphic species exhibited higher intraspecific spectral variability and lower accuracy linked to differences between fertile/sterile fronds than monomorphic species. FT-NIR proves effective as a complementary tool for fern systematics, elucidating species limits and diversity patterns. Further studies should address how hybridization, introgression, and indumentum affect spectral data.

**Supplementary Information:**

The online version contains supplementary material available at 10.1007/s10265-026-01697-1.

## Introduction

The continuous evolution of lineages drives the necessity to develop new approaches to explore biodiversity and define taxonomic boundaries (Hörandl 2022; Wiens 2007). Traditional methods relying solely on macromorphological features constitute emerging challenges to our understanding of biodiversity (Dayrat 2005). Species are often defined using a single line of evidence, which presents significant challenges to understanding evolutionary boundaries (De Queiroz 2007). They are generally not constructed or considered testable hypotheses and are often described in ways that impede their validation (De Queiroz 2007; Sites and Marshall 2003; Wiens 2007). Additionally, plants tend to exhibit greater phenotypic plasticity compared to animals (Borges 2008; Huey et al. 2002; Palacio-López et al. 2015), making it even more challenging to understand the phenotypic complexity of this group.

An accurate and multidimensional definition of species boundaries is crucial for understanding the diversity of living organisms, allowing us to determine whether their definition corresponds or not to a single lineage (Dayrat 2005; Will et al. 2005). This is particularly important since species complexes represent a significant part of biodiversity (Pinheiro et al. 2018). Complexes are lineages that are still incompletely separated and in which reproductive barriers remain lax (Pinheiro et al. 2018). For cryptic species, despite significant genetic divergences, low levels of morphological disparity are observed between lineages, making their identification more difficult (Struck et al. 2018). Integrating data from different sources (e.g., morphology, ecological variation, molecular phylogenetics, etc.) can overcome the limitations of traditional methods (Cheng et al. 2021; Edwards and Knowles 2014; Yeates et al. 2011) based mainly on macromorphological characters that are not always diagnostic and can sometimes be homoplastic (Quattrini et al. 2019). To address this, approaches incorporating data from different levels of biological organization (Dayrat 2005) promote complementarity between fields of study and encourage collaboration among specialists. Emerging methods can help us understand taxonomically recalcitrant groups, advancing our knowledge of biological diversity and the evolutionary processes that shape it (Rouhan and Gaudeul 2021; Sandall et al. 2023).

One method in evidence is Fourier-transform near-infrared spectroscopy (FT-NIR) (Pasquini 2018). This technique is promising due to its speed, accessibility, non-destructive nature, and lack of sample pre-treatment (Rodríguez-Fernández et al. 2011). The fundamental principle of the method involves exposing fragments of biological material (e.g., a dry leaf) to near-infrared radiation (Durgante et al. 2013; Paiva et al. 2021). The spectra generated by the material represent the energy absorbed by the C–H, C–N, and C–O bonds based on the amount of light absorbed (Pasquini 2003, 2018). This process generates complex spectra that allow the sample’s chemical and physical structure to be analyzed (Durgante et al. 2013; Workman and Weyer 2007). Spectroscopy is the tool of choice in a wide range of studies, from those focused on understanding ontogeny (Fernandes et al. 2020) to plant stress (Zahir et al. 2022) and nutrient analysis of leaf tissues (Prananto et al. 2020). It is also used to delimit and discriminate species, genera, and families (Prata et al. 2018; Xu et al. 2009), as well as for the recognition of new plant species (Gaem et al. 2020; Vasconcelos et al. 2021). In the context of spectral studies on ferns, only one study has been conducted so far specifically on this lineage, aiming to explore the method’s efficiency (Paiva et al. 2021).

Ferns, the second most diverse group of vascular land plants (Nitta et al. 2022), are widely distributed around the world (Suissa et al. 2021). Their greatest diversity is found in montane tropical regions (Suissa et al. 2021). These plants, which lack flowers and fruits, have a life cycle characterized by alternating generations, including both sporophyte and gametophytic phases (Haufler et al. 2016). Since many lineages show cryptic variations (Ekrt et al. 2022; Kinosian et al. 2020; Wei et al. 2022; Yi et al. 2023), frequent hybridization events (Bloesch et al. 2022; Luo et al. 2024; Mendez-Reneau et al. 2024), and/or polyploidy (Fujiwara et al. 2023; Heslop-Harrison et al. 2023), it has proven challenging to delimit species based solely on morphological analyses. Both polyploidy and hybridization are important and are crucial factors in speciation (Alix et al. 2017; Wood et al. 2009). Hybridization, particularly, is contributing to the emergence of new lineages and adding complexity to evolutionary processes (Sigel 2016).

**Fig. 1:**
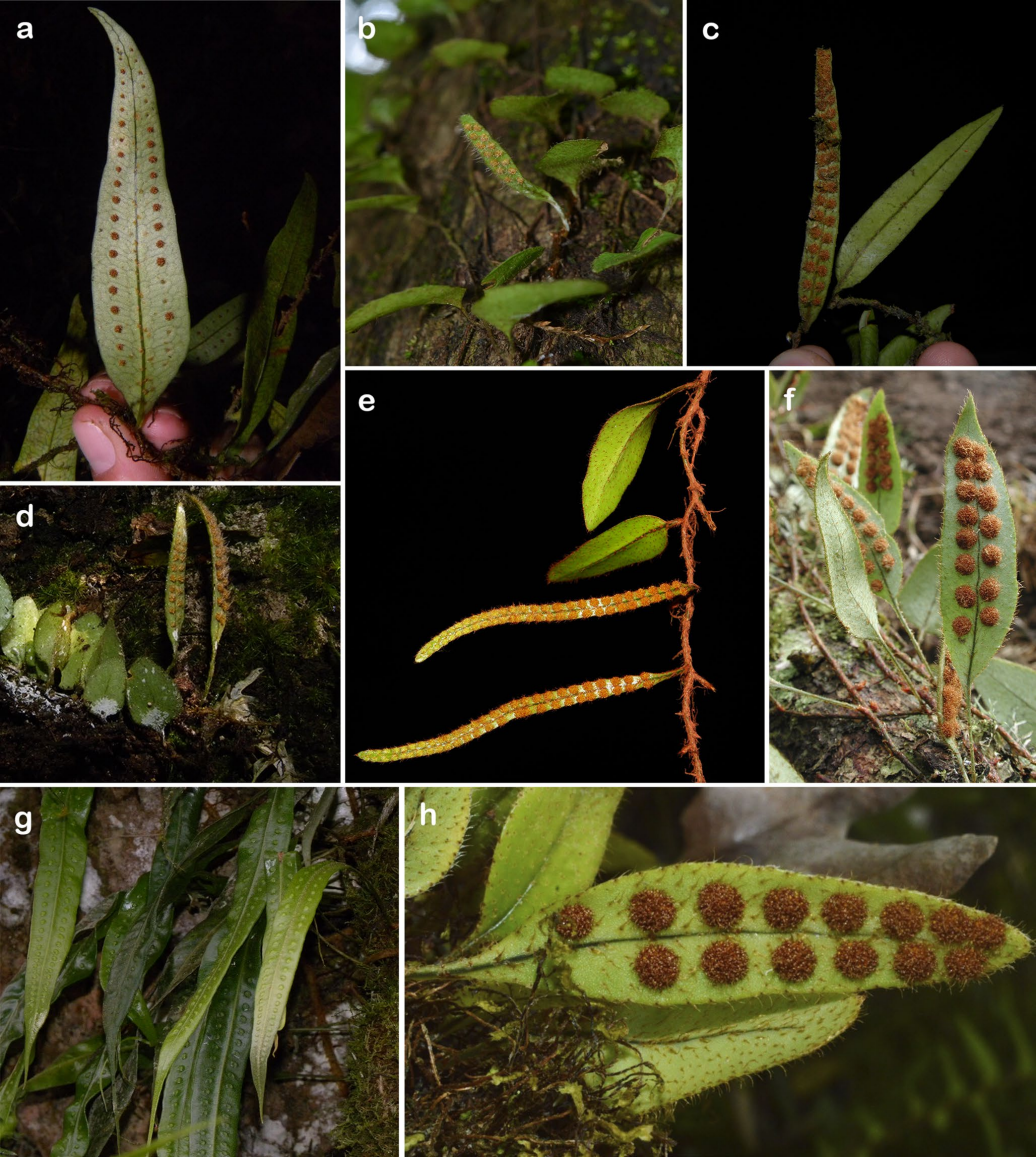
*Microgramma* species from the Scaly clade (polypodiaceae). **a** *M. dictyophylla*. **b** *M. nana*. **c** *M. tobagensis*. **d** *M. tecta*. **e** *M. reptans* (© by 2016 C.N. Fraga). **f** *M. latevagans* (© by 2009 M. Sundue). **g** *M. percussa*. **h** *M. piloselloides* (© by 2018 A. Jose)

Our study focuses on the Scaly clade of the fern genus *Microgramma* C.Presl (Polypodiaceae), whose main synapomorphy is the presence of subulate and/or round scales on both sides of the frond surfaces (sensu Almeida et al. 2021). This clade is sister to the remainder of the genus and diverged ca. 26 mya (Almeida et al. 2021). With a distribution that covers the entire American tropical region, it includes five robust, well-supported species with distinct morphological traits (*M. dictyophylla* (Kunze ex Mett.) de la Sota, *M. latevagans* (Maxon & C.Chr.) Lellinger, *M. nana* (Liebm.) T.E.Almeida, *M. percussa* (Cav.) de la Sota, and *M. tecta* (Kaulf.) Alston), and three variable species with overlapping morphology (*M. piloselloides* (L.) Copel., *M. reptans* (Cav.) A.R.Sm., and *M. tobagensis* (C.Chr.) C.D.Adams & Baksh.-Com.), which hinder taxonomic identification. Some species are widely distributed, such as *M. dictyophylla*,* M. nana*, *M. percussa*, *M. reptans*, and *M. tobagensis*, while others have restricted distributions, such as *M. piloselloides* (Central America and the Caribbean), or are microendemic, such as *M. latevagans* (Bolivia and Peru) and *M. tecta* (Brazilian Atlantic Forest) (Almeida et al. 2021). The morphological disparity in the clade is also significant, with some species exhibiting frond dimorphism (*M. nana*,* M. reptans*,* M. tecta*, and *M. tobagensis*) while others are monomorphic (*M. latevagans*,* M. piloselloides*,* M. dictyophylla*, and *M. percussa*) (Almeida 2014; Almeida et al. 2021) (Fig. 1).

The phylogenetic inference by Almeida et al. (2021) revealed potential issues in the circumscription of one species within the Scaly clade. *Microgramma M. tobagensis*, which has a disjunct distribution between the Andes+northern South America, and the easternmost part of Brazil, was recovered as polyphyletic. A sample from Peru clustered with *M. piloselloides*, while a specimen from eastern Brazil grouped with *M. reptans* (Almeida et al. 2021). Additionally, *M. nana* and *M. tecta*, previously treated as a variety of the same species, were not recovered as sister lineages (Almeida et al. 2021). Their close morphological similarity represents evolutionary convergence, highlighting how homoplasy in traditional morphological traits can confound species delimitation. This underscores the value of additional tools like spectral analysis for studying challenging complexes.

Using a spectral approach, this study evaluates how near-infrared spectroscopy performs across species with contrasting taxonomic robustness using the Scaly clade of the fern genus *Microgramma*. We address the following questions: How do spectral signatures differ between well-circumscribed species and taxonomically problematic species (showing morphological overlap or indications of polyphyly)? Are there differences in the spectral structure between monomorphic and dimorphic species, and fertile and sterile fronds? To this end, we tested the following hypotheses: (I) near-infrared spectral signatures will have higher accuracy distinguishing between well-circumscribed taxa and lower accuracy for taxa with documented phenotypic variation, character overlap (sensu Almeida 2014) and suggestions of non-monophyly (Almeida et al. 2021); (II) there are significant differences between the spectra of fertile and sterile fronds in dimorphic ferns and less variation in monomorphic ferns.

## Materials and methods

### Sampling

We analyzed dry herbarium specimens from the following collections: BHCB, INPA, MO, and NY (acronyms according to Thiers 2025; continuously updated). We analyzed 94 samples from eight species within the Scaly clade (Fig. 1; Table S1), representing geographical and morphological variation within the clade. We followed a strict protocol to select fronds for spectral readings, avoiding young, damaged, and brittle fronds with the presence of fungi and other epiphyllous organisms. All the specimens analyzed in this study were identified by the senior author, following the species circumscription of Almeida (2014). To test the difference in accuracy between fertile and sterile fronds in monomorphic and dimorphic species, we captured the spectra of three fertile fronds and three sterile fronds from each specimen, whenever available.

### Spectral signature

**Fig. 2:**
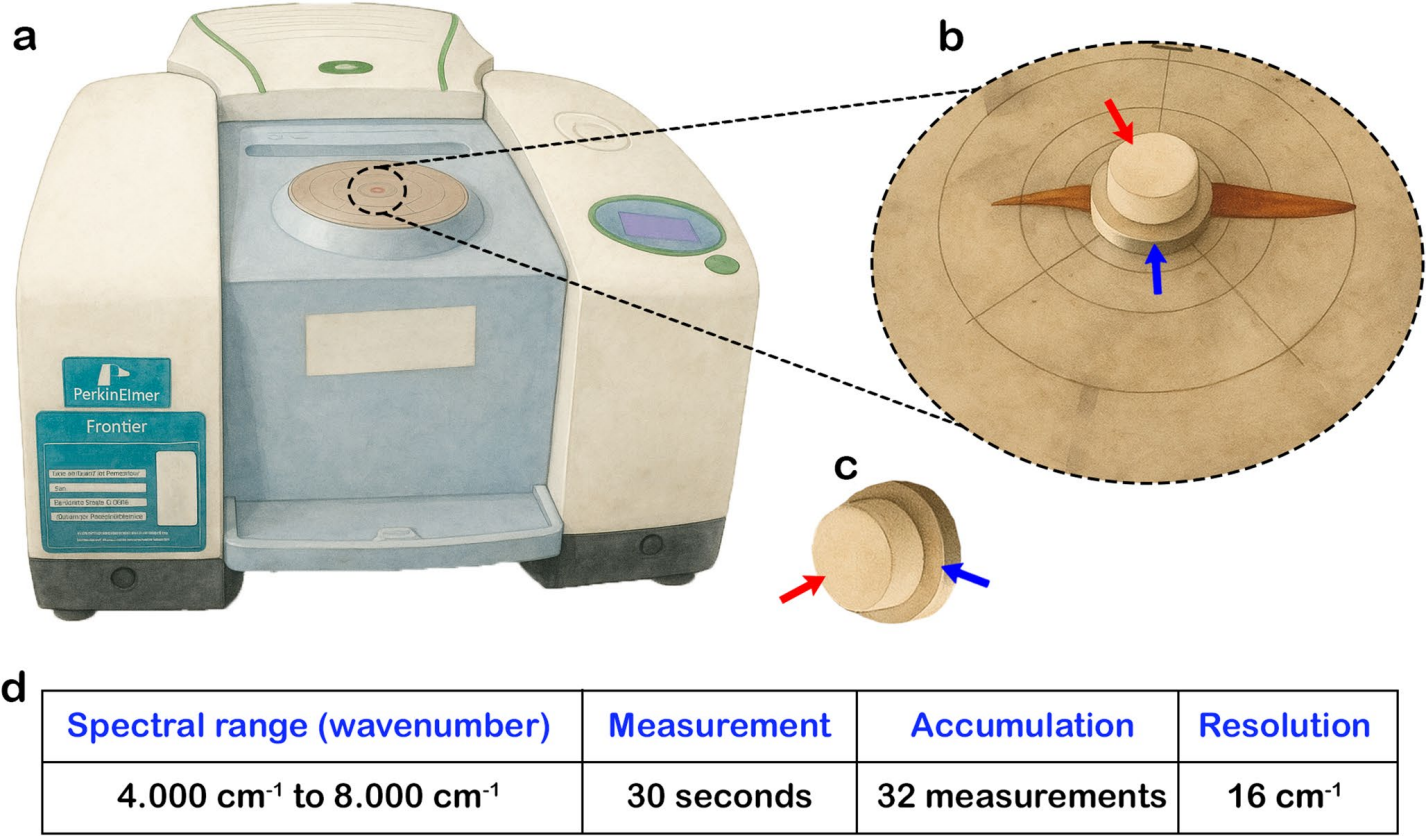
FT-NIR spectroscopy equipment and measurement settings used in this study. **a** PerkinElmer Frontier™ spectrometer. **b** Close-up of the beam aperture showing the reference Sphere over a fern frond. **c** Reference sphere showing the dual-surface design. **d** Settings used in the equipment for spectral readings. White Spectralon surface (red arrows) used for calibration and metallic surface (blue arrows) used during frond measurements to minimize light scattering

We collected 1882 spectral readings in absorbance values, two from each frond surface (abaxial and adaxial), using a PerkinElmer Frontier™ Fourier Transform near-infrared (FT-NIR) spectrometer (Fig. 2a, b). Before reading each sample, we performed a background calibration using a white reference sphere made of Spectralon, a fluoropolymer with high reflectance and low absorption (Fig. 2c). To avoid light scattering effects, we positioned the metallic side of the Spectralon over the fronds during spectrum capture. Each measurement lasted 30 s, with 32 accumulation measurements to ensure the accuracy and quality of all spectra, at a resolution of 16 cm^− 1^, totaling 2,001 absorbance values per measurement, in the spectral range from 4.000 cm^− 1^ to 8.000 cm^− 1^ (wavenumber), equivalent to 1.250 nm to 2.500 nm (wavelength) (Fig. 2d).

The equipment has an automatic performance verification (APV) system, which reduces the frequency of systematic calibrations by continually monitoring performance and alerting to the need for adjustments. All raw data were collected in an environment with a constant and controlled temperature of 18 °C.

### Data pre-processing

After collection, the .txt files generated by the FTNIR spectrometer were processed and compiled in a data table using the *readr* package. Subsequently, we performed initial statistical tests to ensure the accuracy and consistency of the spectral data. Any discrepant values (outliers) were visually identified and manually removed from the dataset (non-standard peaks, lines without waves compatible with the expected chemical profile of a frond, or excess noise). Additionally, we applied standardization using the Standard Normal Variate (SNV) method. This method involves adjusting each spectrum by subtracting the mean and dividing by the standard deviation of each data point (Barnes et al. 1989). This process increases the comparability of the spectra by eliminating dispersion effects and variations in absolute intensity (Barnes et al. 1989). Normalization of spectra using the SNV method was performed using the base R functions ‘mean’ and ‘sd’ (Mailund 2019; R Core Team 2024).

### Data analysis

We created three models for the spectral tests: Dataset (I) evaluated spectra from fertile fronds only; Dataset (II) investigated spectra from sterile fronds only; and Dataset (III) used a combination of data from fertile and sterile fronds. The data were prepared and manipulated using the *dplyr* package (Mailund 2019). To explore the variation in raw spectral data in multidimensional space, we performed Principal Component Analysis (PCA) (Kherif and Latypova 2020) using the *stats* package (R Core Team 2024), analyzing all individual readings.

To test spectral variation across fertile and sterile fronds within each species and between-species within each frond type (fertile vs. sterile), we performed a three-way ANOVA using the stats package (R Core Team 2024). Initial assumption testing revealed violations of normality (Shapiro-Wilk test on model residuals: *p* = 0.020; Shapiro and Wilk 1965). We implemented a Generalized Linear Model (GLM) with a Gamma distribution via the *stats* package (McCullagh 2019; R Core Team 2024). We assessed term significance using Type III hypothesis tests (implemented in the *car* package; Fox and Weisberg 2019). For post-hoc comparisons between factor levels, we conducted pairwise contrasts with Tukey-adjusted *p-values* using the *emmeans* package (Lenth et al. 2023).

To test informativeness and accuracy of each model, Partial least squares discriminant analysis (PLS-DA) was performed for each model using the *pls* package (Mevik and Cederkvist 2004), within the framework provided by the *caret* package (Kuhn 2008). This analysis involves categorizing samples based on predefined categories, focusing on identifying components that effectively account for variations among the variables in different classes while disregarding noise and uncorrelated variations (Mevik and Cederkvist 2004). We selected PLS-DA for discriminant analysis because it does not require the assumptions of normality and homogeneity of covariances (Hastie et al. 2009a), which are often violated in spectral data and biological samples. To account for within-specimen variation while avoiding pseudo-replication, for this analysis, we averaged all spectra readings separately for fertile and sterile fronds from each specimen.

To evaluate model performance and generalizability, we implemented two cross-validation approaches. In the first, K-fold cross-validation, the dataset was randomly partitioned into K equal subsets. In each of K iterations, the model was trained on K–1 subsets and validated on the remaining subset, with performance metrics averaged across all iterations. This provides a robust estimate of the model’s accuracy across the entire dataset (Burman 1989; Yadav and Shukla 2016). Additionally, we used the Leave-One-Out Cross-Validation (LOOCV), a special case of K-fold validation where K equals the total number of samples (N). Each iteration used N-1 samples for training and one for validation, providing a less biased but computationally intensive assessment (Kohavi 1995). K-fold validation was used because it is computationally more efficient and reduces variance in performance estimates (Hastie et al. 2009b). LOOCV, on the other hand, was applied for a more detailed assessment of model sensitivity, especially in contexts with reduced sample sizes (Hastie et al. 2009b). To prevent data leakage and artificial overestimation of accuracy, all readings from the same individual were grouped within the same subset, ensuring independence between the training and testing sets. Additionally, we evaluated how data pre-processing approaches affected model accuracy for all combinations of models and cross-validation methods. We compared three data treatments: (1) raw data, (2) outlier removal, and (3) outlier removal and spectra processed with Standard Normal Variate (SNV) transformation. All analyses were performed in R version 4.2.3 (R Core Team 2024).

## Results

**Fig. 3:**
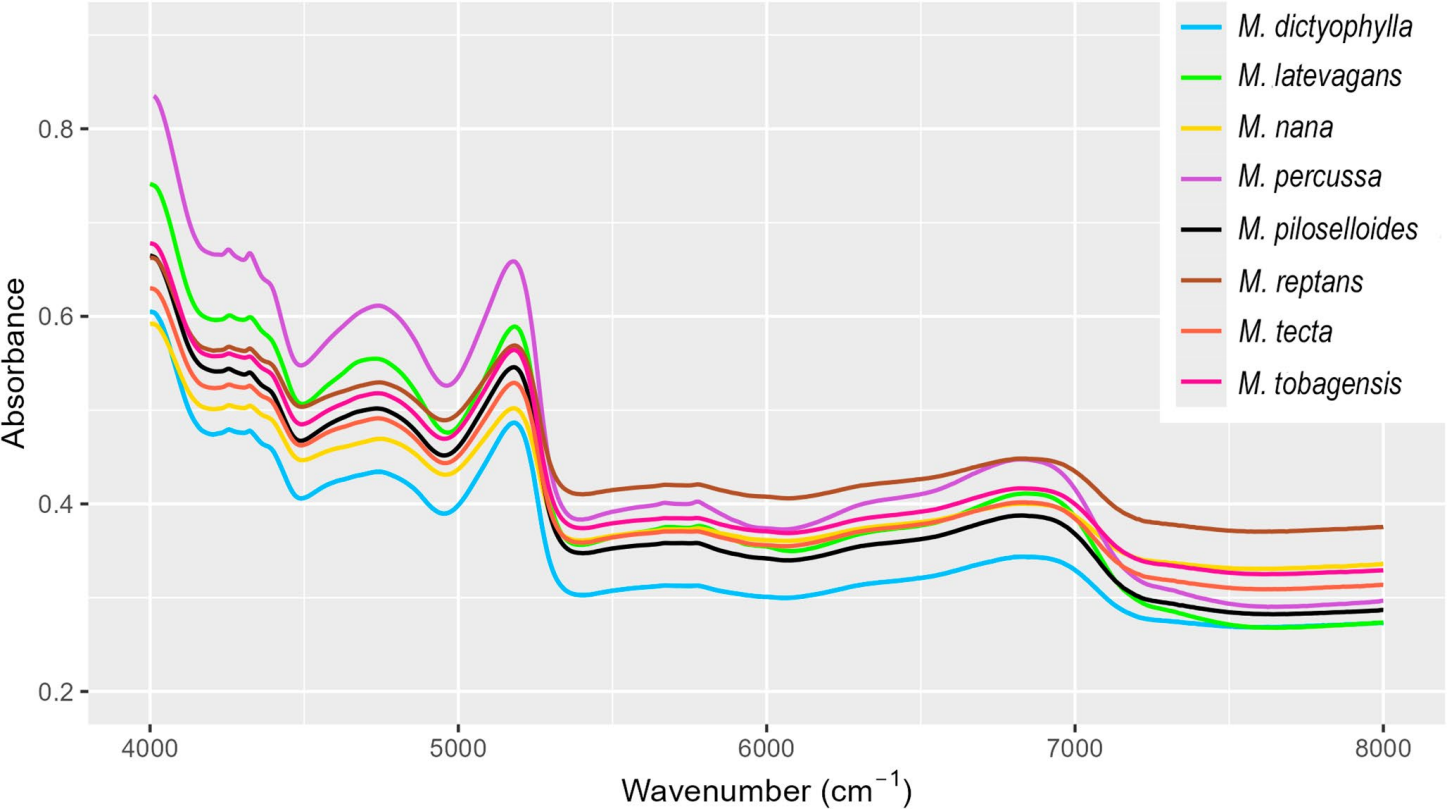
Spectral signature (average data) for eight *Microgramma* species from the Scaly clade

**Fig. 4:**
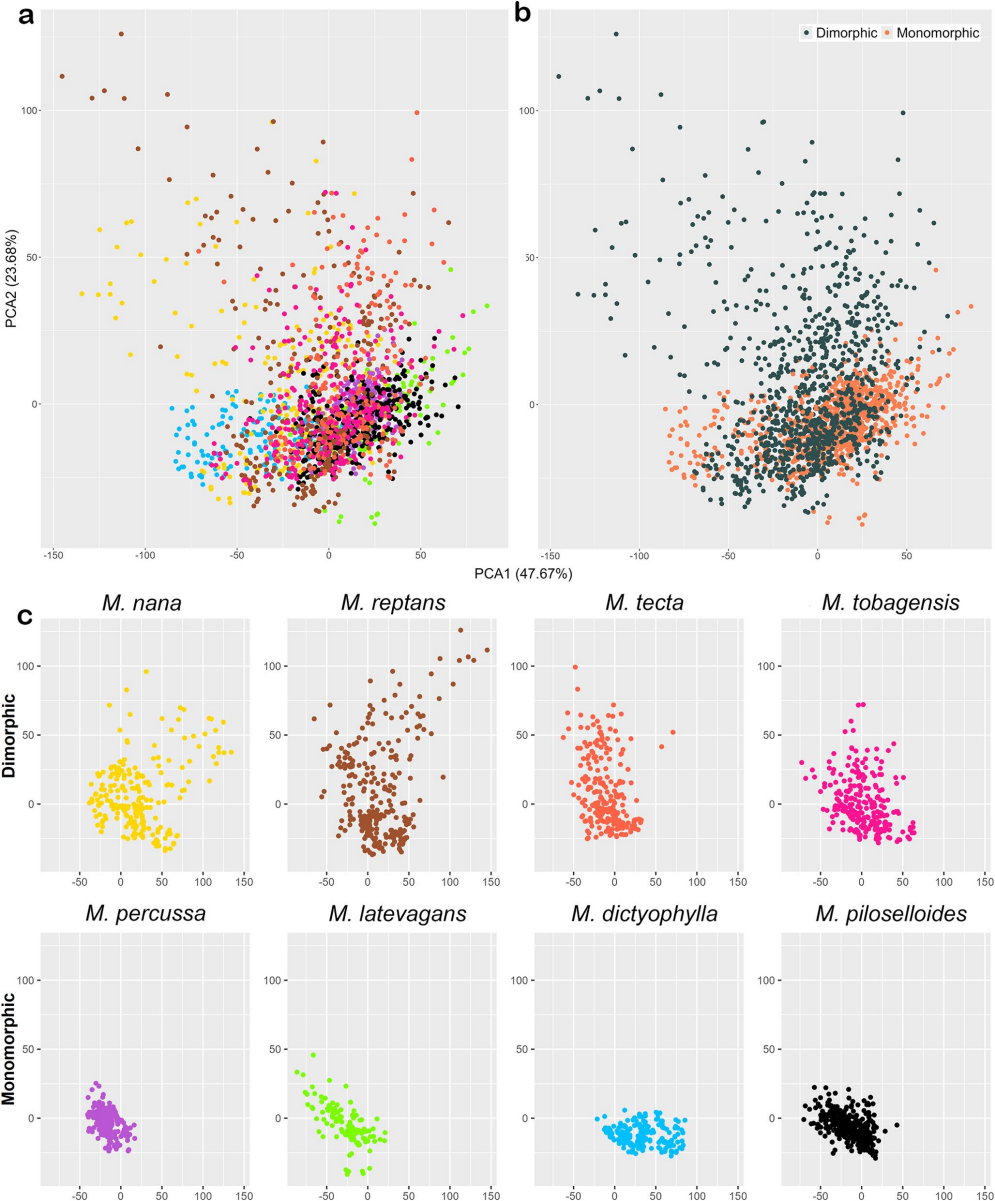
Principal component analysis (PCA). **a** Complete dataset showing the eight tested species: *M. dictyophylla*, *M. latevagans*, *M. nana*, *M. percussa*, *M. piloselloides*, *M. reptans*, and *M. tobagensis*. **b** Specimens categorized by frond dimorphism (dimorphic and monomorphic). **c** Same PCA, showing species separately

The average spectral signatures exhibited absorbance curves with similar shapes among the lineages (Fig. 3), differing mainly in the overall magnitude of absorbance, which indicates variation in signal intensity, and not in the spectral profile itself. The PCA including all species (dataset III) captured 71.3% of the spectral variation in the first two axes (PCA1 = 47.7%, PCA2 = 23.7%), with considerable overlap between species (Fig. 4a). The same overlap was observed when exploring dimorphic and monomorphic species separately (Fig. 4b). Spectral variation is higher in dimorphic than monomorphic species (Fig. 4b), where dimorphic species (*M. nana*,* M. tecta*,* M. reptans*,* and M. tobagensis*) showed greater variation than monomorphic species (*M. percussa*,* M. latevagans*,* M. dictyophylla*, and *M. piloselloides*) (Fig. 4b). This divergence is best observed in the PCA of each species, to compare which species are most overlapping (Fig. 4c).

**Fig. 5:**
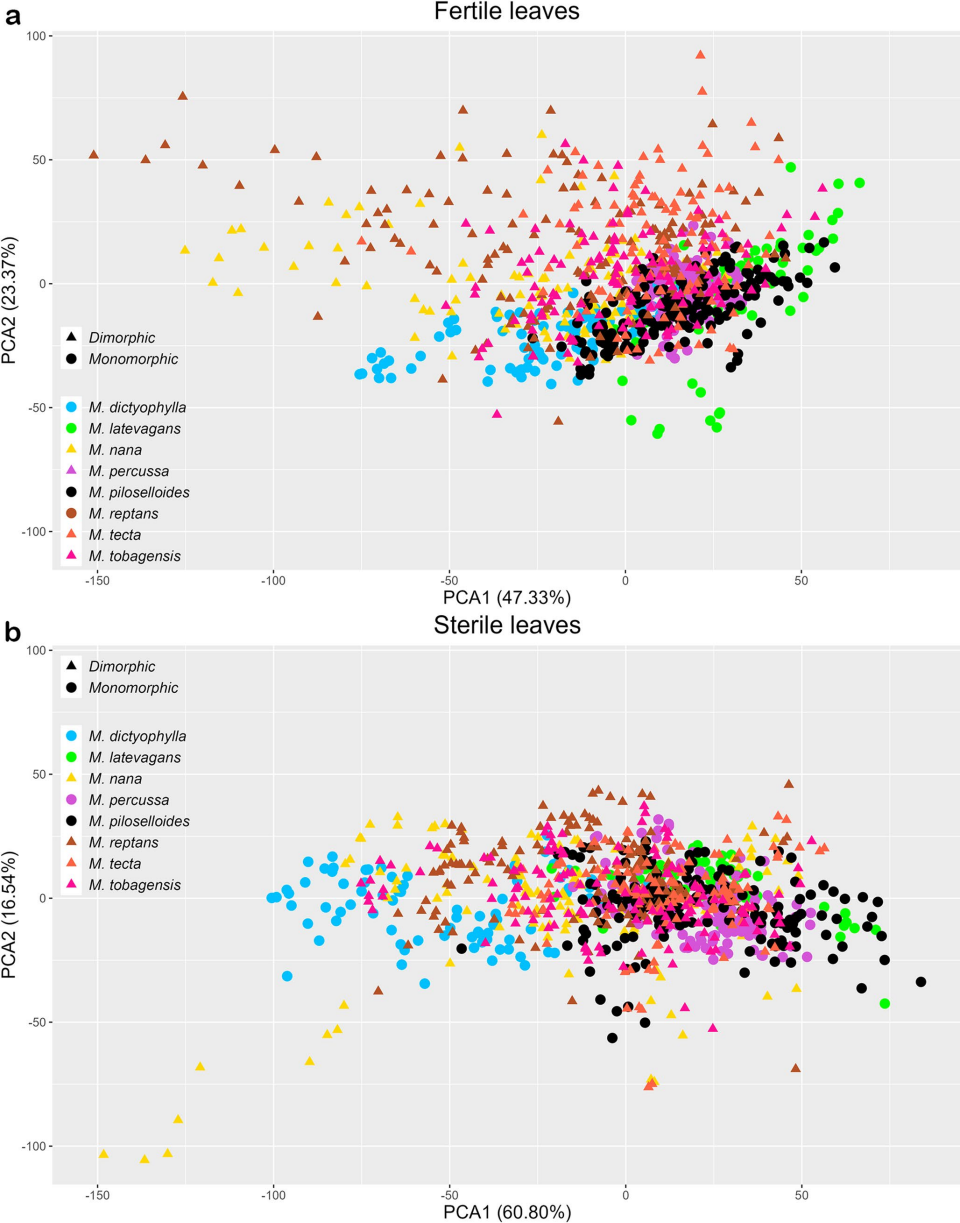
Principal component analysis (PCA). **a** Fertile frond spectra across all eight *Microgramma* species. **b** Sterile frond spectra across all eight *Microgramma* species

In the PCA of dataset I, using data from fertile fronds only (Fig. 5a), 70.6% of the spectral variation was captured by the first two axes (PCA1 = 47.3%, PCA2 = 23.3%). In dataset II, using sterile fronds (Fig. 5b), the first two axes captured 77.3% of the variation (PCA1 = 60.8%, PCA2 = 16.5%). The spectral overlap between species was clearly visible in both datasets I and II (Fig. 5a, b). The occupation of spectral space by fertile (Dataset I) and sterile (Dataset II) fronds varied among species. For *M. dictyophylla*, *M. nana*, and *M. percussa*, fertile fronds (Dataset I) occupied a smaller spectral space than sterile fronds (Dataset II). In contrast, *M. reptans* showed the opposite pattern, with fertile fronds (Dataset I) occupying a broader spectral space than sterile fronds (Dataset II). The spectral space occupation in *M. latevagans*, *M. piloselloides*, and *M. tecta* did not differ between fertile and sterile fronds (Fig. 5a, b).

**Fig. 6:**
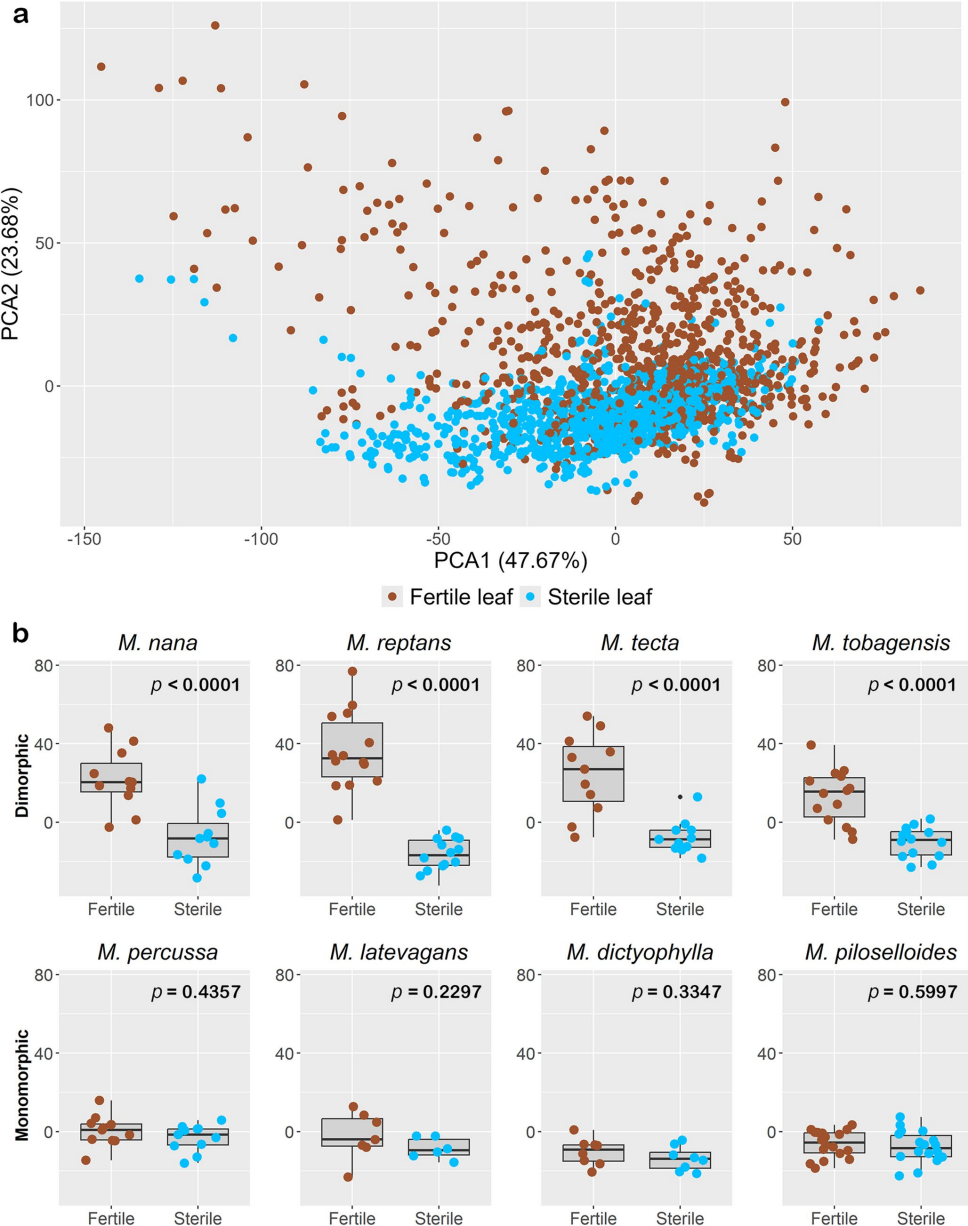
Principal component analysis (PCA). **a** Mean FT-NIR spectra for all eight species, grouped by frond type (fertile, red; sterile, blue). **b** Box plots comparing fertile vs. sterile frond spectra for each species, with significance levels from pairwise GLM comparisons (Tables S2-S3, for full statistics). Dimorphic species (upper row) show greater frond-type divergence than monomorphic species (lower row). The two figures are represented by the same x and y axes of the PCA

Fertile fronds showed broader spectral variation than sterile ones (Fig. 6a). When analyzed separately (Fig. 6b), dimorphic species (*M. nana*,* M. reptans*,* M. tecta*, and *M. tobagensis*) presented significantly (*p* < 0.0001) higher spectral disparity between fertile and sterile fronds compared to monomorphic species (*M. percussa*,* M. latevagans*,* M. dictyophylla*, *and M. piloselloides* (Fig. 6b; Table S2). Fertile fronds showed significant spectral differentiation between monomorphic and dimorphic species (Table S3), with two exceptions: *M. latevagans* vs. *M. tobagensis* (*p* = 0.185) and *M. percussa* vs. *M. tobagensis* (*p* = 0.2896; Table S3).

**Fig. 7:**
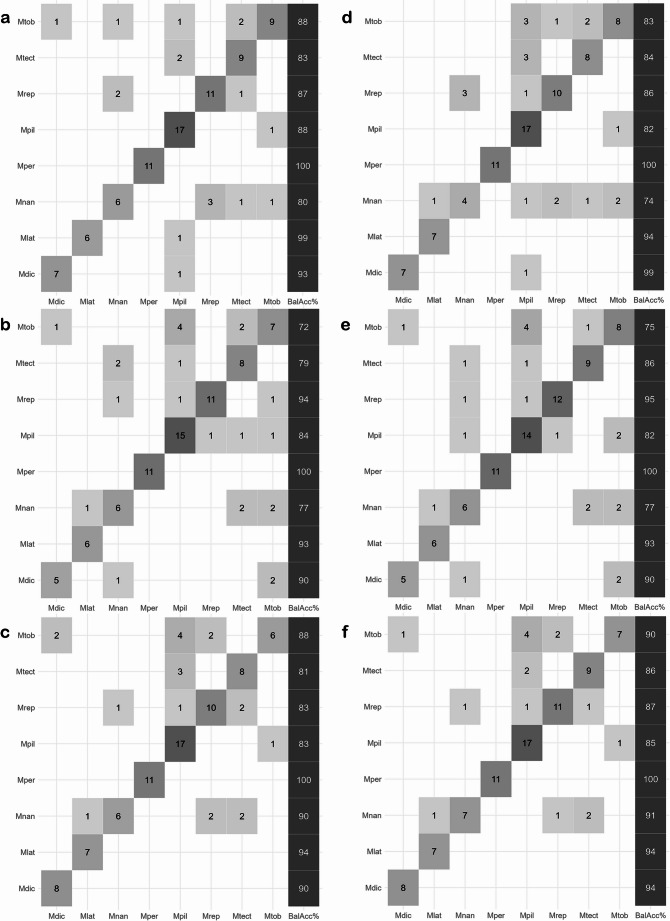
Confusion matrices resulting from partial least squares discriminant analysis (PLS-DA) for K-fold and LOOCV validations. **a** K-fold validation for fertile fronds. **b** K-fold validation for sterile fronds. **c** K-fold validation for combined fronds. **d** LOOCV validation for fertile fronds. **e** LOOCV validation for sterile fronds. **f** LOOCV validation for combined fronds. The values on the diagonal represent the number of correctly predicted species, while the off-diagonal values indicate the number of prediction errors. The last column shows the balanced accuracy value for each species. The abbreviations correspond to the names of the *Microgramma* species: Mtob = *M. tobagensis*, Mtect = *M. tecta*, Mrep = *M. reptans*, Mpil = *M. piloselloides*, Mper = *M. percussa*, Mnan = *M. nana*, Mlat = *M. latevagans*, Mdic = *M. dictyophylla*

In the discriminant analyses of the three tested models (Dataset I - fertile fronds only; Dataset II - sterile fronds only; and Dataset III - both fertile and sterile fronds), monomorphic species outperformed dimorphic ones (Table 1). Among the monomorphic species, *M. percussa* stood out with 100% prediction accuracy in all models for both validations (Fig. 7; Table 2). *Microgramma latevagans* and *M. dictyophylla* also performed well, each achieving a prediction accuracy higher than 90%. Among monomorphic species, *M. piloselloides* had the lowest accuracy rates, ranging from 82% to 88% (Fig. 7; Table 2). Despite this, it still outperformed many of the dimorphic species.

**Table 1:** Accuracy (%) of models tested in validations and confidence interval (CI%) before and after pre-processing

Data	Raw data		Outlier removal		Outlier removal + Standard Normal Variate (SNV)		Monomorphic species only		Dimorphic species only	
Models/Validation	K-fold	LOOCV	K-fold	LOOCV	K-fold	LOOCV	K-fold	LOOCV	K-fold	LOOCV
Fertile	77% (69–85%)	77% (69–84%)	82% (73–89%)	81% (73–88%)	81% (71–88%)	77% (67–85%)	95% (84–99%)	98% (88–100%)	76% (61–87%)	73% (57–86%)
Sterile	75% (66–84%)	78% (70–86%)	73% (65–82%)	75% (66–83%)	74% (64–83%)	76% (66–84%)	100% (90–100%)	100% (92–100%)	76% (60–88%)	83% (69–93%)
both	77% (69–84%)	76% (68–84%)	77% (69–85%)	77% (68–85%)	78% (68–86)	82% (73–89%)	100% (92–100%)	100% (90–100%)	77% (61–89%)	83% (68–93%)

values ​​between “(….)” are equivalent to the Confidence Interval (CI%) and above these values ​​the accuracy for each model and validation tested

**Table 2:** Number of species and hit rates (balanced accuracy%) for the tested models and functional types of fronds of the scaly *Microgramma* clade (Polypodiaceae)

Species	Number of species	Model I - fertile		Model II - sterile		Model III - both		Average accuracy values
		K-fold	LOOCV	K-fold	LOOCV	K-fold	LOOCV	
Monomorphic	45	–	–	–	–	–	–	–
*M. dictyophylla*	08	93%	99%	90%	90%	90%	94%	93%
*M. latevagans*	07	99%	94%	93%	93%	94%	94%	94%
*M. percussa*	11	100%	100%	100%	100%	100%	100%	100%
*M. piloselloides*	19	88%	82%	84%	82%	83%	85%	84%
Dimorphic	49	–	–	–	–	–	–	–
*M. reptans*	14	87%	86%	94%	95%	83%	87%	89%
*M. nana*	11	80%	74%	77%	77%	90%	91%	81%
*M. tecta*	11	83%	84%	79%	86%	81%	86%	83%
*M. tobagensis*	13	88%	83%	72%	75%	88%	90%	83%

Among the dimorphic species, *M. reptans* performed best, with accuracy values ranging from 83% to 95%. *Microgramma tecta* also displayed good results, ranging from 79% to 86% (Fig. 7; Table 2). *Microgramma nana* showed a wide variation, between 74% and 91%, while *M. tobagensis* ranged between 72% and 90% accuracy (Fig. 7; Table 2). Although the dimorphic species showed varied performances, none reached the optimum accuracy achieved by *M. percussa*, which received 100% of the predictions.

Pre-processing with outlier removal combined with Standard Normal Variate (SNV) correction (Table 1) enhanced model accuracy compared to datasets that only had outliers removed, particularly for Datasets II and III. Dataset I, which included fertile fronds only, showed a slight reduction in accuracy after the pre-processing, from 82% to 81% using K-fold cross-validation and from 81% to 77% using leave-one-out cross-validation (LOOCV). Despite this minor reduction, pre-processed data still outperformed raw data (Table 1). For the sterile fronds model (Dataset II), pre-processing the data resulted in a slight positive change in accuracy. In the K-fold cross-validation, accuracy increased from 73% to 74%, representing a difference of 1.4%, and LOOCV showed an increase from 75% to 76%, with a 1.3% improvement. The combined model (Dataset III) also improved considerably, with K-fold accuracy increasing from 77% to 78%, a gain of 1.3%, while in the LOOCV, accuracy increased from 77% to 82%, representing a 6.5% improvement.

## Discussion

This study is the first to investigate the spectral behavior of functionally different photosynthetic organs, such as the dimorphic fronds of ferns, and to evaluate the efficiency of this tool in a clade of ferns with recognized species circumscription complexities (Almeida et al. 2021). In the pioneering study by Paiva et al. (2021), the FT-NIR method demonstrated high accuracy in identifying fern species. Our results corroborate the usefulness of FT-NIR even in species with difficult taxonomic delimitation. The accuracy of species discrimination depends on the construction of robust spectral models (Vieira et al. 2021), which, in turn, reflect well-defined circumscriptions, as observed in some species in this study. In addition to species circumscription, variations in the spectral signature of a species can be affected by environmental conditions (e.g., temperature, humidity) (Xu et al. 2019), soil properties (Asner et al. 2014), and developmental stages, such as differences between juvenile and adult fronds due to their chemical composition (Lang et al. 2015; Neuwirthová et al. 2021).

In some species of the Scaly clade of the genus *Microgramma*, morphological overlap makes it difficult to precisely define and identify the individuals studied (Almeida 2014). The average percentage of correct identifications for our three models (sterile fronds, fertile fronds, and both) ranged from 81% to 100% based on the validation methods we used, with an overall average of 88% correct identifications. Only three species (*M. tecta*, *M. nana*, and *M. tobagensis*) had accuracy percentages below 80%.

Heteroblasty and heterophylly are widespread among land plants and have ecological, evolutionary, and taxonomic importance (Zotz et al. 2011). The spectral data suggest that dimorphic species exhibit significant intra-individual spectral variation. Our results also suggest that, when considering both fertile and sterile fronds indistinctively, they have lower prediction percentages when compared to monomorphic species (Fig. 6; Table 1). This relates to the functional and morphological duality of the fronds of dimorphic species (Vasco et al. 2013; Wagner and Wagner 1977; Watkins et al. 2016), which may introduce higher intrinsic variability into the spectral signature of these species. In contrast, monomorphic species have a single frond morphotype that performs all functions (Vasco et al. 2013), resulting in less spectral variability within an individual and, therefore, better predictive accuracy.

In fertile fronds, the existence of sori on the abaxial surface (Wagner and Wagner 1977) did not appear to compromise model accuracy, as our models presented similar and higher accuracy levels in Dataset I (Fertile) and Dataset III (Both) (Table 1), a hypothesis that should be tested formally in future studies. However, when considering monomorphic and dimorphic species separately, the models continue to have similar accuracies among the three datasets tested (Table 1). Most FT-NIR studies suggest that models such as Dataset III that combine different data sets, in this case, fertile and sterile fronds, are the best predictive models due to their comprehensive assessment of spectral signatures. For example, Paiva et al. (2021) obtained better results with the combination of abaxial and adaxial data. Similarly, Lang et al. (2015) obtained better prediction results by combining data from fronds of young and adult trees. Our results indicate a somewhat similar, but still variable, perspective. Dataset III is better in dimorphic species, while in monomorphic species, the accuracies are equal between Datasets II and III (Table 1). When considering the species together, Datasets I and III present the highest accuracies (Table 1).

The species *M. percussa* and *M. reptans* obtained highly accurate results, with 100% and 89%, respectively (Table 2). This result was unexpected, given that these species occupy a variety of habitats ranging from open areas to forests, growing on rocks and as epiphytes from lower trunks to the canopy (Almeida 2014). The fronds of *M. percussa* also present a wide variation in width, length, and shape — a variation that, together with their wide distribution in the American tropics, from Mexico to southern Brazil, is reflected in its ten heterotypic synonyms (Almeida 2014). For *M. reptans* the results are unexpected because this species presents a wide variation in the shape of the sterile fronds, with sterile specimens being hard to distinguish from *M. tobagensis*, for example (Almeida 2014). *Microgramma dictyophylla* and *M. latevagans* also showed high identification accuracy rates, with 93% and 94%, respectively. Although they are morphologically distinct, with variations in frond shape and size, frond surface indumentum, and spore type (Almeida 2014), their distribution areas partially overlap (Almeida 2014; Lima et al. unpublished manuscript). *Microgramma latevagans* is endemic to Bolivia and Peru, while *M. dictyophylla* has a wider distribution, covering northern South America. However, they do not occur sympatrically, as *M. latevagans* is found at high elevations (above 2,000 m.a.s.l.), while *M. dictyophylla* grows in lowland forests. Furthermore, the high overall accuracy of their spectral models may reflect good circumscriptions, favored by the specific and striking morphological characteristics of each of them. The few errors in the discriminant analysis related to these two models did not occur between these two species (Fig. 7).

Two morphologically similar species, *M. tobagensis* and *M. piloselloides*, obtained 83% and 84% correct predictions in the PLS-DA (Table 2). The errors might have different explanations. First, the identifications might not be correct, as there is considerable overlap in some of the characteristics used to identify them (e.g., indument and shape of fertile and sterile fronds). Although they mostly do not occur sympatrically (Almeida et al. 2021; Smith et al. 2018), there are populations of *M. piloselloides*,* M. tobagensis*, and *M. reptans* co-occurring in Guatemala and Costa Rica (Almeida 2014; Lima et al. unpublished manuscript). Our results showed one incorrect prediction of *M. reptans* as *M. tobagensis* and five incorrect predictions of *M. tobagensis* as *M. reptans* (Fig. 7). The most striking differences between these species are found in the sterile fronds; several sterile specimens of *M. tobagensis* are difficult to distinguish from *M. reptans*. On the other hand, phylogenetic data suggest the polyphyly of *M. tobagensis* (Almeida et al. 2021), a conflicting circumscription that may be reflected in the observed spectral errors (Fig. 7). In our investigations, two specimens initially identified as *M.* cf. *tobagensis* were predicted as *M. piloselloides*. After revisiting these specimens, we found they were misidentified and were confirmed as *M. piloselloides*. This correction significantly improved the accuracy of the models. We believe that processes such as hybridization and introgression might be playing a role in the evolution of these populations. Adding to the phylogenetic evidence presented by Almeida et al. (2021), spectral data suggest that further studies should focus on a broad sampling of these species populations to test their circumscription.

*Microgramma tecta* and *M. nana*, which are morphologically similar (Almeida et al. 2021), also had lower predictive identification accuracy. *Microgramma tecta* had an average accuracy of 83% and *M. nana* 81%, considering all models and validation (Table 2). We believe that the small size of the fertile and sterile fronds, along with the high coverage of trichomes and scales, may have influenced the spectral accuracy. However, no tests have yet been conducted to determine the influence of indument on spectral readings. A fundamental aspect to be considered is the geographic overlap of *M. nana* with other species, such as *M. reptans*, *M. tobagensis*, and *M. dictyophylla*, which raises the possibility of hybridization and introgression between these lineages. Natural hybridization is well documented in vascular plants, especially between phylogenetically close species that co-occur (Liao et al. 2015; Wu et al. 2024). In these sympatry zones, where two or more species coexist under similar environmental conditions, the probability of interspecific crosses increases, generating viable hybrids with a combination of phenotypic traits from the parental species (Sawangproh et al. 2020). Furthermore, introgression – when hybrids backcross with their parental species – can promote the exchange of adaptive genes between populations, increasing genetic variability and potentially favoring local adaptation (Li et al. 2021; Suarez-Gonzalez et al. 2018). These processes, by introducing new genetic traits, can impact the morphological and chemical responses of plants (López-Caamal and Tovar-Sánchez 2014), creating additional variability between populations. This genetic and phenotypic complexity can, theoretically, influence the effectiveness of spectral predictive models. Further studies are needed to determine exactly how hybridization and introgression affect the results.

It is important to consider that the robustness of the models may vary according to the specific context of each study and the characteristics of the species analyzed. Despite testing separate models for fertile and sterile fronds, the morphological variability between these fronds (Watkins et al. 2016) results in different spectral responses in dimorphic species compared to monomorphic ones. This variation occurs because fertile and sterile fronds are subject to additional variations arising from factors such as stage of development, light exposure, and environmental conditions (Dalgallo Rocha et al. 2013; Moran 1987). This performance underscores the effectiveness and high quality of monomorphic species in the context of our analysis, as evidenced by their higher performance. However, the lower accuracy of dimorphic species does not necessarily diminish their relevance or usability in studies using spectral data. It reflects the complexity and variability associated with the morphofunctional frond duality of these species. We believe that when including dimorphic and monomorphic species in the same set of discriminant and validation data, controlling these variations separately can significantly improve the overall accuracy results of the models, as observed in our results (Table 1). Given the diversity of heteroblasty found among ferns (Vasco et al. 2013), an expanded sampling to encompass other lineages is necessary to test whether the pattern we found here is relevant to other groups.

## Conclusions

This study offers valuable insights into the accuracy and effectiveness of FT-NIR in capturing intra and interspecific variation in ferns, which have fewer macromorphological characteristics than flowering plants. Our findings highlight the value of spectral data as an informative tool in evidence-based testing of hypotheses in plant systematics. New methodological multidimensional approaches must be sought, in addition to morphological data, especially when dealing with species complexes. The average overall accuracy of 88% was good considering the complexity of the clade. Prediction values are much more meaningful when looking at species individually (Fig. 7; Table 2), such as the 100% accuracy for *M. percussa*. Our results reflect in detail the complex relationships of phylogenetically close species, highlighting the usefulness of spectral data for species discrimination. Finally, we reinforce the importance of a well-planned experimental design for future studies using FT-NIR for other ferns, which should explicitly account for possible spectral variation across fertile and sterile fronds, and dimorphic and monomorphic species. Evaluating and designing effective strategies for utilizing this method will be crucial in employing new approaches and maximizing the potential of spectral data for species discrimination and evolutionary analyses.

## Supplementary Information

Below is the link to the electronic supplementary material.

Supplementary Material 1

## Data Availability

All data and R code used in this study are publicly available in the GitHub repository: https://github.com/labevofern/Microgramma-FTNIR.
