# Supplementary material for: A comparison of interspecific and intraspecific phenotypic variation in spectral signatures of ferns with robust versus uncertain species boundaries
Source: J Plant Res. 2026 Mar 9;139(3):349–64. doi: 10.1007/s10265-026-01697-1 (PMC13197310; doi:10.1007/s10265-026-01697-1)
Supplement: Supplementary file 1 — Supplementary Material 1 [file 10265_2026_1697_MOESM1_ESM.pdf]

Supplementary electronic materials

Title: A comparison of interspecific and intraspecific phenotypic variation in spectral signatures of ferns with robust versus uncertain species boundaries

Authors: Niksoney Azevedo Mendonça, Marise Helen Vale de Oliveira and Thaís Elias Almeida

Journal: Journal of Plant Research

Corresponding author: Niksoney Azevedo Mendonça (Universidade Federal de Pernambuco, Programa de Pós-graduação em Biologia Vegetal).

Phone: +55-98-991213636

E-mail: niksoney.azevedo@ufpe.br

Table S1 – Specimens used for spectral data capture. Herbaria acronym follows Thiers (2025 continually updated: https://sweetgum.nybg.org/science/ih/).

**Species**	**Country**	**Voucher**
Microgramma dictyophylla	Brazil	Vida 739 (BHCB145204)
Microgramma dictyophylla	Brazil	Almeida 2230 (BHCB136581)
Microgramma dictyophylla	Brazil	Almeida 2605 (BHCB144728)
Microgramma dictyophylla	Brazil	Cowan 38507 (NY00880074)
Microgramma dictyophylla	Ecuador	Gilmartin 320 (MO1799919)
Microgramma dictyophylla	Guyana	Maguire 40547 (NY03961499)
Microgramma dictyophylla	Peru	Foster 7419 (MO3232987)

Microgramma dictyophylla	Venezuela	Liesner 24298 (NY03961515)
Microgramma latevagans	Bolivia	Smith 13487 (MO4008613)
Microgramma latevagans	Bolivia	Rodriguez 1263 (MO6145673)
Microgramma latevagans	Bolivia	Antezana 1438 (MO04857892)
Microgramma latevagans	Bolivia	Lewis 881089 (MO4008174)
Microgramma latevagans	Peru	Sánchez Vega 5979 (MO3292118)
Microgramma latevagans	Peru	Dillon 6092 (NY03230430)
Microgramma latevagans	Peru	Bennett 2601 (NY03349971)
Microgramma nana	Brazil	Almeida 2732 (BHCB149816)
Microgramma nana	Brazil	Silva 168 (INPA113289)
Microgramma nana	Brazil	Carvalho UAT83 (INPA226341)
Microgramma nana	Brazil	Almeida 2632 (BHCB144755)
Microgramma nana	Costa Rica	Grayum 4884 (MO3324161)
Microgramma nana	Ecuador	Aulestia 128 (MO6016797)
Microgramma nana	Ecuador	Pérez 1185 (NY03962278)
Microgramma nana	Mexico	Campos Villanueva 1011 (MO5069443)
Microgramma nana	Suriname	Maguire 24002 (MO1312664)
Microgramma nana	Suriname	Herrera 10088 (MO6069412)
Microgramma nana	Venezuela	Davidse 3042 (MO2982712)
Microgramma percussa	Brazil	Fraga 3104 (BHCB150254)
Microgramma percussa	Brazil	Dittrich 842 (BHCB64514)
Microgramma percussa	Brazil	Barreto 2595 (BHCB154070)
Microgramma percussa	Brazil	Almeida 372 (BHCB101385)
Microgramma percussa	Brazil	Salino 1988 (BHCB29357)
Microgramma percussa	Brazil	Madison 618 (INPA85192)

Microgramma percussa	Colombia	Croat 79621 (MO6027011)
Microgramma percussa	Colombia	Luteyn 10469 (NY03961982)
Microgramma percussa	Mexico	Hernández 445 (NY03961228)
Microgramma percussa	Panama	McDaniel 8077 (MO6576856)
Microgramma percussa	Peru	Campos 3991 (MO5302244)
Microgramma piloselloides	Bonaire	Boom 11057 (NY02099903)
Microgramma piloselloides	Costa Rica	Rojas 2067 (MO5900546)
Microgramma piloselloides	Cuba	Underwood 831 (NY01842269)
Microgramma piloselloides	Cuba	Britton 5165 (NY01842276)
Microgramma piloselloides	Dominica	Chambers 2501 (NY01842425)
Microgramma piloselloides	Grenada	Broadway s.n. (MO5446677)
Microgramma piloselloides	Jamaica	s.c/s.n. (MO5462147)
Microgramma piloselloides	Jamaica	s.c/s.n. (MO5462145)
Microgramma piloselloides	Jamaica	Hitchcock s.n. (MO5446665)
Microgramma piloselloides	Jamaica	s.c/s.n. (MO5446680)
Microgramma piloselloides	Puerto Rico	Ahlquist 45 (BHCB173388)
Microgramma piloselloides	Puerto Rico	Britton 2062 (NY00989767)
Microgramma piloselloides	Puerto Rico	Underwood 11 (NY00989773)
Microgramma piloselloides	Puerto Rico	Shafer 3139 (NY00989780)
Microgramma piloselloides	Puerto Rico	Vincent 15355 (NY02150502)
Microgramma piloselloides	Haiti	Nash 217 (NY01842393)
Microgramma piloselloides	Dominican Republic	Mejía 7508 (MO3859988)
Microgramma piloselloides	Dominican Republic	Zanoni 28119 (NY01842360)
Microgramma piloselloides	St Vincent and The Grenadines	Badger s.n. (MO5484127)

Microgramma reptans	Brazil	Daly 1352 (INPA118203)
Microgramma reptans	Brazil	Matos 11468 (CEPEC15838)
Microgramma reptans	Brazil	Fraga 3090 (BHCB150244)
Microgramma reptans	Brazil	Fraga 2971 (BHCB143826)
Microgramma reptans	Colombia	Molina 18c716 (MO1626606)
Microgramma reptans	Costa Rica	Lesica 4136 (MO3416567)
Microgramma reptans	Ecuador	Aulestia 1560 (MO05055185)
Microgramma reptans	Guyana	MC Dowell 4809 (MO04621215)
Microgramma reptans	Nicaragua	Urbina 70 (MO6710204)
Microgramma reptans	Panama	Salino 15879 (BHCB173590)
Microgramma reptans	Panama	Stern 646 (MO1817771)
Microgramma reptans	Panama	Kennedy 3232 (MO3014609)
Microgramma reptans	Peru	Schunke-Vigo 3469 (INPA133051)
Microgramma reptans	Peru	King 451 (INPA123964)
Microgramma tecta	Brazil	Krieger 8862 (BHCB165025)
Microgramma tecta	Brazil	Salino s.n. (BHCB43418)
Microgramma tecta	Brazil	Salino 10145 (BHCB91558)
Microgramma tecta	Brazil	Salino 13779 (BHCB124333)
Microgramma tecta	Brazil	Mota 3160 (BHCB105182)
Microgramma tecta	Brazil	Schmitt 334 (BHCB142994)
Microgramma tecta	Brazil	Kollmann 4051 (BHCB108513)
Microgramma tecta	Brazil	Matos 415 (BHCB97283)
Microgramma tecta	Brazil	Salino 13595 (BHCB122753)
Microgramma tecta	Brazil	Salino 8637 (BHCB81163)
Microgramma tecta	Brazil	Cadorin 2121 (BHCB142972)

Microgramma tobagensis	Brazil	Thomas 9304 (MO04895073)
Microgramma tobagensis	Brazil	Mori 11468 (NY00674449)
Microgramma tobagensis	Brazil	Amorim 4205 (NY01241079)
Microgramma tobagensis	Brazil	Matos 1577 (NY02064119)
Microgramma tobagensis	Ecuador	Gudiño Jara 2256 (MO04911176)
Microgramma tobagensis	Ecuador	Clark 575 (MO5149584)
Microgramma tobagensis	Ecuador	Vargas lópez 5138 (MO6389474)
Microgramma tobagensis	Guatemala	Türckheim 125 (NY03961400)
Microgramma tobagensis	Peru	Klug 2539 (NY03966108)
Microgramma tobagensis	Trinidad and Tobago	Broadway 9959 (NY02027319)
Microgramma tobagensis	Trinidad and Tobago	Jermy 2870 (NY02027320)
Microgramma tobagensis	Trinidad and Tobago	Jermy 2680 (MO04642633)
Microgramma tobagensis	Venezuela	Steyermark 61888 (NY03966137)

Table S2 – Within species comparison of fertile and sterile fronds based on spectral means using a Generalized Linear Model (GLM) with Gamma distribution and logarithmic link function. Results are presented on the natural logarithm scale, with *p-values* adjusted using Tukey's method for multiple comparisons. SE = standard error, df = degrees of freedom, t.ratio = t-Statistic.

**Comparison**	**Species**	**Estimate**	**SE**	**df**	**t.ratio**	***p-value***
Fertile - Sterile leaf	*M. dictyophylla*	0.17094	0.177	171	0.967	0.3347
Fertile - Sterile leaf	*M. latevagans*	0.23698	0.197	171	1.205	0.2297
Fertile - Sterile leaf	*M. nana*	0.77819	0.151	171	5.164	<.0001
Fertile - Sterile leaf	*M. percussa*	0.11773	0.151	171	0.781	0.4357
Fertile - Sterile leaf	*M. piloselloides*	0.06194	0.118	171	0.526	0.5997
Fertile - Sterile leaf	*M. reptans*	1.4882	0.134	171	11.142	<.0001
Fertile - Sterile leaf	*M. tecta*	0.83061	0.151	171	5.512	<.0001
Fertile - Sterile leaf	*M. tobagensis*	0.71927	0.134	171	5.385	<.0001

Table S3 – Between-species pairwise comparisons within each frond type (fertile vs. sterile) based on spectral means using a Generalized Linear Model (GLM) with Gamma distribution and logarithmic link function. Results are presented on the natural logarithm scale, with *p-values* adjusted using Tukey's method for multiple comparisons. SE = standard error, df = degrees of freedom, t.ratio = t-Statistic.

**Comparison**	**Frond type**	**Estimate**	**SE**	**df**	**t.ratio**	***p-value***
M. dictyophylla - M. latevagans	Fertile	-0.31197	0.183	171	-1.706	0.6836
M. dictyophylla - M. nana	Fertile	-0.90001	0.164	171	-5.481	<.0001
M. dictyophylla - M. percussa	Fertile	-0.39773	0.164	171	-2.422	0.2375
M. dictyophylla - M. piloselloides	Fertile	-0.16836	0.15	171	-1.121	0.9514
M. dictyophylla - M. reptans	Fertile	-1.1375	0.157	171	-7.263	<.0001
M. dictyophylla - M. tecta	Fertile	-0.95346	0.164	171	-5.806	<.0001
M. dictyophylla - M. tobagensis	Fertile	-0.7279	0.157	171	-4.647	0.0002
M. latevagans - M. nana	Fertile	-0.58804	0.171	171	-3.442	0.0163
M. latevagans - M. percussa	Fertile	-0.08576	0.171	171	-0.502	0.9996
M. latevagans - M. piloselloides	Fertile	0.14361	0.157	171	0.912	0.9846
M. latevagans - M. reptans	Fertile	-0.82553	0.164	171	-5.046	<.0001
M. latevagans - M. tecta	Fertile	-0.64149	0.171	171	-3.754	0.0057
M. latevagans - M. tobagensis	Fertile	-0.41593	0.164	171	-2.543	0.1850
M. nana - M. percussa	Fertile	0.50228	0.151	171	3.333	0.0229
M. nana - M. piloselloides	Fertile	0.73164	0.135	171	5.41	<.0001
M. nana - M. reptans	Fertile	-0.23749	0.142	171	-1.668	0.7078
M. nana - M. tecta	Fertile	-0.05345	0.151	171	-0.355	1.0000
M. nana - M. tobagensis	Fertile	0.17211	0.142	171	1.209	0.9284
M. percussa - M. piloselloides	Fertile	0.22937	0.135	171	1.696	0.6899
M. percussa - M. reptans	Fertile	-0.73977	0.142	171	-5.196	<.0001
M. percussa - M. tecta	Fertile	-0.55572	0.151	171	-3.688	0.0072
M. percussa - M. tobagensis	Fertile	-0.33017	0.142	171	-2.319	0.2896
M. piloselloides - M. reptans	Fertile	-0.96914	0.126	171	-7.696	<.0001
M. piloselloides - M. tecta	Fertile	-0.78509	0.135	171	-5.805	<.0001
M. piloselloides - M. tobagensis	Fertile	-0.55954	0.126	171	-4.443	0.0004
M. reptans - M. tecta	Fertile	0.18404	0.142	171	1.293	0.9006

M. reptans - M. tobagensis	Fertile	0.4096	0.134	171	3.067	0.0503
M. tecta - M. tobagensis	Fertile	0.22556	0.142	171	1.584	0.7592
M. dictyophylla - M. latevagans	Sterile	-0.24593	0.191	171	-1.289	0.9020
M. dictyophylla - M. nana	Sterile	-0.29276	0.164	171	-1.783	0.6325
M. dictyophylla - M. percussa	Sterile	-0.45094	0.164	171	-2.746	0.1162
M. dictyophylla - M. piloselloides	Sterile	-0.27737	0.15	171	-1.847	0.5890
M. dictyophylla - M. reptans	Sterile	0.17976	0.157	171	1.148	0.9451
M. dictyophylla - M. tecta	Sterile	-0.29379	0.164	171	-1.789	0.6283
M. dictyophylla - M. tobagensis	Sterile	-0.17957	0.157	171	-1.147	0.9454
M. latevagans - M. nana	Sterile	-0.04683	0.179	171	-0.261	1.0000
M. latevagans - M. percussa	Sterile	-0.20501	0.179	171	-1.143	0.9462
M. latevagans - M. piloselloides	Sterile	-0.03143	0.167	171	-0.189	1.0000
M. latevagans - M. reptans	Sterile	0.42569	0.172	171	2.469	0.2162
M. latevagans - M. tecta	Sterile	-0.04786	0.179	171	-0.267	1.0000
M. latevagans - M. tobagensis	Sterile	0.06636	0.172	171	0.385	0.9999
M. nana - M. percussa	Sterile	-0.15818	0.151	171	-1.05	0.9659
M. nana - M. piloselloides	Sterile	0.0154	0.135	171	0.114	1.0000
M. nana - M. reptans	Sterile	0.47252	0.142	171	3.319	0.0240
M. nana - M. tecta	Sterile	-0.00103	0.151	171	-0.007	1.0000
M. nana - M. tobagensis	Sterile	0.11319	0.142	171	0.795	0.9932
M. percussa - M. piloselloides	Sterile	0.17357	0.135	171	1.283	0.9039
M. percussa - M. reptans	Sterile	0.6307	0.142	171	4.43	0.0004
M. percussa - M. tecta	Sterile	0.15715	0.151	171	1.043	0.9671
M. percussa - M. tobagensis	Sterile	0.27137	0.142	171	1.906	0.5488
M. piloselloides - M. reptans	Sterile	0.45713	0.126	171	3.63	0.0088
M. piloselloides - M. tecta	Sterile	-0.01642	0.135	171	-0.121	1.0000
M. piloselloides - M. tobagensis	Sterile	0.0978	0.126	171	0.777	0.9941
M. reptans - M. tecta	Sterile	-0.47355	0.142	171	-3.326	0.0235
M. reptans - M. tobagensis	Sterile	-0.35933	0.134	171	-2.69	0.1327
M. tecta - M. tobagensis	Sterile	0.11422	0.142	171	0.802	0.9928
